# Therapeutic drug repositioning with special emphasis on neurodegenerative diseases: Threats and issues

**DOI:** 10.3389/fphar.2022.1007315

**Published:** 2022-10-03

**Authors:** Bibhuti Bhusan Kakoti, Rajashri Bezbaruah, Nasima Ahmed

**Affiliations:** Department of Pharmaceutical Sciences, Faculty of Science and Engineering, Dibrugarh University, Dibrugarh, India

**Keywords:** drug repurposing, alzheimer, parkinson, neurodegenarative disease, artificial intelligence

## Abstract

Drug repositioning or repurposing is the process of discovering leading-edge indications for authorized or declined/abandoned molecules for use in different diseases. This approach revitalizes the traditional drug discovery method by revealing new therapeutic applications for existing drugs. There are numerous studies available that highlight the triumph of several drugs as repurposed therapeutics. For example, sildenafil to aspirin, thalidomide to adalimumab, and so on. Millions of people worldwide are affected by neurodegenerative diseases. According to a 2021 report, the Alzheimer’s disease Association estimates that 6.2 million Americans are detected with Alzheimer’s disease. By 2030, approximately 1.2 million people in the United States possibly acquire Parkinson’s disease. Drugs that act on a single molecular target benefit people suffering from neurodegenerative diseases. Current pharmacological approaches, on the other hand, are constrained in their capacity to unquestionably alter the course of the disease and provide patients with inadequate and momentary benefits. Drug repositioning–based approaches appear to be very pertinent, expense- and time-reducing strategies for the enhancement of medicinal opportunities for such diseases in the current era. Kinase inhibitors, for example, which were developed for various oncology indications, demonstrated significant neuroprotective effects in neurodegenerative diseases. This review expounds on the classical and recent examples of drug repositioning at various stages of drug development, with a special focus on neurodegenerative disorders and the aspects of threats and issues viz. the regulatory, scientific, and economic aspects.

## 1 Introduction

There has been a relentless search for the discovery of drugs in various therapeutic segments. Of late repurposing also referred to as drug repositioning has gained interest in recent years. As per the reports, various discoveries have taken place in the finding of new molecules and the development of alternative strategies using repurposing strategies. In comparison to the classical drug discovery process, the new approach of Drug repurposing (DR) has various advantages and has opened new vistas in the field of Pharmacology and Medicinal chemistry. Treatment of rare and intractable diseases, minimizing attrition rates, reducing the cost of therapy, etc. are some of the advantages of drug repurposing. Essentially it is a new way of approaching drug compounds and targets that have been abandoned during the development stages either to their risks or other issues. This review shed light on the classical and recent examples of DR at various stages of drug development, with a special focus on neurodegenerative diseases (NDs) and the aspects of threats and issues viz. the regulatory, scientific, and economic aspects.

## 2 Drug repurposing approaches

As stated by the U.S. Census Bureau, the world’s population on 1st January 2022 was estimated to be 7.8 billion. This depicts that there is an expansion of 74 million people or a 0.9% growth rate (The Economic Times, 2021). Furthermore, there has been an escalation in the figure of geriatric people that is supplementing the world population growth. The dwellers of each country in the world are enduring build-up in both the proportion and size of elderly persons. It is envisioned that 1 in every 6 people in the globe will be in the age group of 60 years or beyond by 2030. In developed countries, life expectancy is ascending in small doses above 80 years. While there is a deviation in the assortment of a country’s population towards older ages, the frequency of incidence and progression of incurable ND has heightened. Aging is the leading risk factor for nearly all ND including Alzheimer’s disease (AD) and Parkinson’s disease (PD) (Hou et al., 2019). The number of people being afflicted by AD is anticipated to surge up to 135 million by 2050 because AD alone can affect between one-third and one-half of people above the age of 85 years. NDs are expected to have disastrous repercussions on individuals, families, and societies unless efficient aids are discovered to minimize the progression of these diseases.

Over the past century, NDs have generated distinctive and convincing challenges to effective drug discovery. In America, AD and PD are the two uttermost prevalent NDs with 5 million Americans existing with AD as well as more than 500,000 people diagnosed with PD (Karlawish et al., 2017). Yet another group of people comprising millions more are affected with rare NDs, such as amyotrophic lateral sclerosis (ALS), multiple sclerosis (MS), Huntington’s disease (HD), frontotemporal dementia (FTD), and spinal muscular atrophy (Katsnelson et al., 2016; Correale et al., 2017). The healthcare cost of contrasting dementias and AD scores for over US$200 billion, an amount presumably to escalate by 2050 if these disorders persist to be unrecoverable (Barnes, 2021). It has been unveiled that there is no cure for MS even though as many as 9 immunomodulatory compositions have reached FDA approval for MS since 2000. This is shockingly diverse from the instance that even though the number of AD patients is pondered to approximately double in the following 10 years, only four non-disease-modifying compounds were passed for AD during that equivalent period (Crismon, 1994; Cusi et al., 2007; Birks and Evans, 2015; Birks and Harvey, 2018). Besides the overwhelming load of AD and other NDs on our healthcare system touching a bothersome level and unfulfilled efficacious cure, the urgency for the well-timed creation of competent therapies has been increasing bit by bit.

**FIGURE 1:**
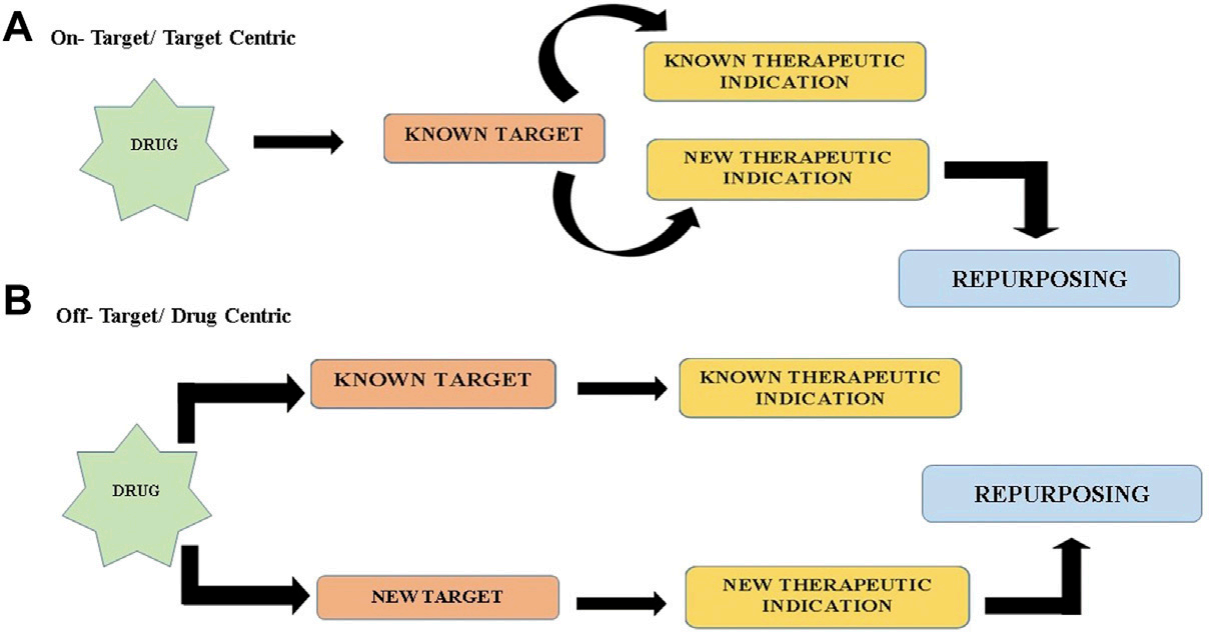
Two cardinal strategies of drug repurposing **(A)** On target/target Centric **(B)** Off target/Drug Centric.

Currently, treatments accessible for NDs can barely handle the symptoms or terminate the progression of the disease (Durães et al., 2018). The drug discovery process right from target identification and validation to licensed use of a drug is a daunting task that comes with a long gestation period. DR (DR) is a present-day trending strategy that overcomes several shortcomings of the denovo development of entirely new drugs. It speeds up the discovery process and is efficient, economical, riskless, and reduces the failure rates in the clinical development and testing phases (Tanoli et al., 2021). With the expanding necessity for the treatment of NDs and the commitment given by DR, it makes sense that old drugs are being used as new treatments for these diseases. Nonetheless, the foremost issue in drug repositioning is tracking down novel drug-disease relationships. To deal with this issue, there are a range of approaches and two cardinal strategies of DR, viz., on-target and off-target (refer Figure 1) (Rudrapal et al., 2020). In on-target (target-centric) DR, the pharmacological mechanism of a drug molecule that is previously established is correlated to a new therapeutic implication. In this plan of action, the biological target of the drug candidate is unaltered, but the ailment is dissimilar. It incorporates computational approaches, biological experimental approaches, and mixed approaches (Ferreira and Andricopulo, 2016). On the other way around, in the off-target (drug-centric) profile, the pharmacological mechanism of a drug candidate is unrecognized. Drugs and drug candidates respond to new targets, out of the original scope, for afresh curative indications. Consequently, the targets along with the indications are unique (Ashburn and Thor, 2004). In the sphere of DR emphasis is given to three significant stages: procreation of candidate compounds, preclinical analysis, and clinical trial. For the production of candidate compounds, it’s of high priority to determine relevant drugs for potential remedial indications. Notable advances have been made in the understanding of neurodegerative disease biology. Likewise, a plethora of fresh accessible resources has simplified drug discovery attempts through the medium of drug reprofiling. These incorporate bounteous data from clinical, mechanistic and epidemiological research, development of biomarkers, and a number of well-validated models, both cell and animal-based. Nowadays, the most prevailing drug reprofiling approaches in NDs are predominantly grounded on ad hoc clinical and epidemiological risk assessment in human testing and preclinical alterations in rodent models (Ashburn and Thor, 2004). However, for the accomplishment of superior DR in NDs, more precise and standardized approaches for both activity-based and computational methods should be put into effect. In conjunction with swift advancement in the scientific study of disease, the accessibility to contrasting sophisticated tools available in genomics and bioinformatics and assured clinical drug libraries will immeasurably hasten and promote future endeavours in neurodegenerative disease drug repositioning. For exploration of novel therapeutic liabilities for neurodegenerative disease, two alternative and complementary approaches perhaps be applied widely, one is activity-based/experiment based phenotypic screening and the other is theoretical/in silico-based/computational approaches (Rudrapal et al., 2020). DR can also be approached through a combination of both fields.

### 2.1 Experiment-based approaches

When it comes to the series of actions in drug discovery and drug repurposing, the experiment-based/experimental screening approaches are frequently supposed to be the fundamental step. It refers to the identification of original compounds for new pharmacological utilization entrenched on experimental assays. It necessarily blends protein target-based and cell/organism-based screens *in vitro* and/or *in vivo* disease models without necessitating the employment of every structural data of biological target proteins. In this approach, structural data of target proteins as well as the drug-induced cell/disease phenotypic information is not mandatory. The activity-based approach is also time and labor-consuming and amid the screening process, the generation of false positive hits is low. Experimental repositioning comprises a handful of approaches, essentially the cell assay approach, target screening approach, animal model approach, and clinical approach (Lionta et al., 2014; Oprea and Overington, 2015).

Affinity chromatography and mass spectrometry are two broadly operated proteomic techniques in analyzing drug candidates (Brehmer et al., 2005). In the present age, drug target analysis along with drug repositioning are entangled. DR is distinctive from drug discovery in terms of modification of drug targets. The affinity of drug ligands can be predicted using a cellular thermal stability assay which can map the contact patterns of intracellular targets (Molina et al., 2013). Utilizing this method, a considerable number of molecular on and off-targets have been divulged for numerous clinically approved drugs. New biological targets of well-known drugs are derived *via* affinity matrices chiefly observed in the area of kinases (Klaeger et al., 2016; Scott et al., 2016).

### 2.2 *In-silico* approaches

To accomplish effective therapies for neurodegenerative disease and get the therapies to the clinic faster, computational drug repurposing, or the in silico screening of FDA-approved compounds is advantageous. For investigating drug-target binding kinetics and drug residence times of prevailing drugs or drug candidates, using the computer as assistance for molecular docking is a notable approach (De Benedetti and Fanelli, 2018). In silico/computational drug reprofiling, simulated screening of public databases of mountainous drug/chemical libraries is executed by adopting computational biology and bioinformatics/cheminformatics tools. In this approach, the potential bioactive molecules are identified based on the molecular interaction between the drug molecule and protein target (Talevi, 2018). This calls for structural data of target proteins and drug-induced cell/disease phenotypic data. In-silico based approach is time and labor efficient and has a higher rate of false positive hits during the screening.

For many neurodegenerative disorders, it should be considered that drugs look for satisfactory penetration into the blood-brain barrier (BBB). The two sections for curative means of accessing brain targeting are invasive and non-invasive categories (Alam et al., 2010; Gabathuler, 2010). The invasive category encompasses the transitory rise in BBB permeability, and the non-invasive category primarily engages in the transformation of drug molecules *via* a physiological, chemical, or colloidal carrier system approach. Simultaneously, these methods are also connected with computational approaches.

Lately, the amalgamation of economically feasible large-scale computational capacity with high-throughput clinical, molecular, and structural biology technologies has constructed a modernistic and favorable circumstance to logically repurpose conventional drugs by adopting computational frameworks rather than chance findings. Currently available computational approaches/strategies to DR can be branched into molecular, clinical, and structure-based (biophysical) methods.

Intending to conclude drugs that may modify disease gene marks, molecular approaches have opted which aims to match the drug-gene expression marks pre-and post-drug treatment with disease gene expression marks. It does not depend on prior recognition of the target molecule for high-throughput screening of existing compounds. Currently, resources such as CMap (Connectivity Map) and LINCS are limited in the case of neurodegenerative disease. Molecular approaches of computational drug repositioning integrate genetic, epigenetic, proteomic, transcriptomic and metabolomics evidence to determine promising and up-to-date indications for drugs. Additionally, techniques such as network integration, correlating gene expression profiles amidst a disease model and drug-treated condition, prediction of drug-protein interactions, and implementation of genotype-phenotype associations are also being practiced (Yang and Agarwal, 2011; Chen et al., 2017; Luo et al., 2017). There is an enormous demand for the generation of databases based on transcriptomic drug perturbation in CNS tissues to ascertain the drug response to inappropriate tissue and cell types for neurodegenerative disease. Recently, for AD (AD), a proteotranscriptomic-based computational drug repositioning method named Drug Repositioning Perturbation Score/Class (DRPS/C) resulted based on inverse associations between disease-induced or drug-induced gene and protein perturbation patterns (Lee et al., 2020). Another such instance in the matter of ND is the work by Zhang et al. where the National Human Genome Research Institute-European Bioinformatics Institute Genome-Wide Association Study catalog, PubMed, and the Human Metabolome database were precisely extracted to generate an assembly of proteomic, metabolomics, and genetic signatures of AD (Zhang et al., 2016; Wishart et al., 2018; Buniello et al., 2019). By commixing this multi-omics data with the Therapeutic Target database and Drug Bank drug-target databases, the authors of the study were capable of illustrating a list of 75 drug predictions in AD (Wishart et al., 2006; Li et al., 2018).

In clinical methods of drug discovery and repurposing, large-scale health data such as the electronic medical record (EMR), insurance claims data, clinical trial data, health registries, health surveys, and personal genome testing companies are engaged as a supreme asset. Mount Sinai BioMe cohort and the eMERGE network are two notable illustrations of EMR databases. Meticulous medicine approaches can be utilized with the aid of an abundant sample size. It is effortless to identify drugs that are efficacious in indications other than the primary drug use by taking the patient medication history as an asset. For instance, the latest reconsideration of human trials and Medicare pharmacy claim specifics has recommended that when compared to nonuser counterparts, statin users experience a lower incidence of AD (Geifman et al., 2017). Likewise, utilizing EMR laboratory testing data from Ajou University a group of researchers compared the ‘clinical signatures’ or laboratory test values of patients before drug administration and following drug administration and found two therapies for Kawasaki syndrome that is terbutaline sulfate and ursodeoxycholic acid evoked identical changes in laboratory values. Correlating the disease pairs disclosed that there is a significant extent of resemblance in clinical signatures between Kawasaki syndrome and Amyloid lateral sclerosis (ALS), advocating that terbutaline sulfate can be competent in treating ALS besides Kawasaki syndrome. One of the shortcomings of clinical methods is that before analysis clinical data must be changed into a structured database. Moreover, EMR evidence is oftentimes inadequate and cluttered. In the event of neurodegenerative disease patients are to be longitudinally outlined and for NDs with lengthy disease courses it's strenuous to track the physical and mental wellness and consequences. Also for genetic subtype-specific drug repurposing, clinical data should be paired with genetic data.

However, substantial improvement has been made in the computerized recovery of knowledge from unstructured EMR data (Ford et al., 2016; Delespierre et al., 2017). Recently, Observational Medical Outcomes Partnership (OMOP), has been simulated by the Observation Health Data Sciences and Informatics program. OMOP is a universally accepted scheme to transform claimed information and EMR record data into a uniform and consistent data format with familiar data representations essentially terminologies, coding schemes, etc. (Hripcsak et al., 2015). As a result of mutable data coding and formatting, consecutive statistical analyses can be intended with the slightest information loss. There are alternative linkage procedures that include probabilistic matching strategies and ‘fuzzy’ matching techniques and these techniques take advantage of multiple field values to compare records even when no single field is an exact match (Dean et al., 2001; Malin and Sweeney, 2005).

In biophysical methods, drug-target predictions can be accomplished by taking biochemical characteristics of drugs into accounts such as binding affinity or biophysical properties like 3D conformation (Holdgate et al., 2013; March-Vila et al., 2017). These methods comprise structural, ligand-based, and molecular docking methods and possibly be principally advantageous in NDs such as HD with well-established targets (Nance, 2017). Structural methods utilize the complete advantage of 3D protein configuration data to determine structurally identical drugs that might conceal similar targets (March-Vila et al., 2017). Structural methods employ local site similarity metrics to describe protein binding sites or those that identify two protein environments that can bind the same ligand that is chemiosmotic protein environments (de Franchi et al., 2010; Jalencas and Mestres, 2013). If the hypothesis is such that two diseases share similar target proteins, then a structurally similar molecule/drug may be dynamically useful in both diseases. This can be illustrated by the fact that patients with AD and HD both have marked extra synaptic NR2B subunit-containing N-Methyl-D-aspartate receptors (NMDARs) and increased phosphorylation of NMDARs (Song et al., 2003; Hoe et al., 2009). Establishing drugs that hinder the extra synaptic NMDAR activity using addressing structurally analogous ligands or binding sites depicts a credible strategy for DR in both of these conditions (Ehrnhoefer et al., 2012).

Ligand-based methods presume that two molecules may share similar targets if they share a similar bioactivity profile. To verify innovative targets for conventional drugs/compounds, ligand-based methods pay attention to chemical and biological knowledge such as binding affinity; cellular activity; absorption, distribution, metabolism, and excretion data (Gregori-Puigjane and Mestres, 2008; March-Vila et al., 2017). Ligand-based methods entrust public bioactivity databases such as PubChem, DrugBank, and ChEMBL in opposition to structure-based methods, Docking-based methods implement molecular docking simulations either to predict promising drugs for a given target or novel targets for existing drugs (Kitchen et al., 2004). One such example of docking-based repurposing is to single out droperidol as an established drug in AD by the application of high-throughput ligand–protein inverse docking due to droperidol’s high binding affinity to seven AD target proteins (Xie et al., 2016). Although biophysical methods are competent in drug repositioning, they look for prior labeling of target molecules and demand crystallographic evidence of target and drug molecules.

In recent years, several companies are developing and elaborated Artificial intelligence (AI) and machine learning (ML) based frameworks for drug discovery. These methods are exceptionally proficient at linking diverse classes of data. There has been a blooming diversion towards the evolvement of ML techniques to efficaciously dig for transcriptomic, structural, and clinical data (Mani et al., 2012; Kadurin et al., 2017; Shameer et al., 2017; Butler et al., 2018; Wang et al., 2018; Smith et al., 2019). IBM adopted AI-based text-mining approaches to constitute a semantic model of ALS-associated RNA-binding proteins that may exemplify drug targets. BM could uncover potential ALS-associated RNA-binding by application of this model to a new set of RNA-binding proteins (Bakkar et al., 2018).

#### 2.2.1 Artificial intelligence/machine learning algorithms

In recent years, several companies are developing and elaborated Artificial intelligence (AI) and machine learning (ML) based frameworks for drug discovery. These methods are exceptionally proficient at linking diverse classes of data. There has been a blooming diversion towards the evolvement of ML techniques to efficaciously dig for transcriptomic (Wang et al., 2018; Smith et al., 2019), structural (Kadurin et al., 2017; Butler et al., 2018; Popova et al., 2018), and clinical (Shameer et al., 2017; Nemati et al., 2018). ML is one of the forms of artificial intelligence. It does facilitate vigorous interrogation of multiple datasets by using statistical techniques to determine formerly undetected associations and patterns in the data and in the recent past the approaches have been presenting promising outcomes when applied to drug repurposing of neurodegenerative diseases (Myszczynska et al., 2020). Machine learning algorithms are chiefly classified into supervised, unsupervised and reinforcement learning approaches (Bharadwaj et al., 2021). The ongoing methods most frequently applied to neurodegenerative disease-related data are the supervised machine learning algorithms. IBM adopted AI-based text-mining approaches to constitute a semantic model of ALS-associated RNA-binding proteins that may exemplify drug targets. BM could uncover potential ALS-associated RNA-binding by application of this model to a new set of RNA-binding proteins (Bakkar et al., 2018). In a study, a novel computational approach was reported to predict drug repositioning grounded on a ML algorithm and data integration. The approach in the study relied on the persistent analysis of classification mismatches as genuine reclassifications opportunities. The definiteness of the results were of high levels and were rational with several literature reports (Napolitano et al., 2013). In another study, a novel method “PREDICT” was presented which was based on the observation that drugs that are similar can also be indicated for similar disease (Gottlieb et al., 2011). The method obtained tremendous specificity and sensitivity, more desirable than the existing methods in predicting the large-scale drug indications for both approved drugs and novel molecules. In recent years, it has been a remarkable preference to pave the way for novel computational approaches and deep learning (DL) methods is one such example which commits to intensify the capableness of drug repurposing methods. Approaches known as deep neural networks (DNNs) are adopted by DL which encompasses artificial neural networks including plentiful hidden layers between the input and output layers (Ma et al., 2015). One instance of a work that selected deep learning was in which the authors confirmed how DNN trained on gigantic transcriptional response datasets can assort different drugs to therapeutic categories solely established on their transcriptional profile (Aliper et al., 2016). Additionally, favourable outcomes were obtained by means of a deep learning-based algorithmic framework termed as DeepDTIs (Drug target interaction) which ascertained drug-target interactions using chemical structures and known interactions. (Wen et al., 2017).

#### 2.2.2 Network-based methods

By virtue of immense present-day progress in the sphere of system biology has led to the progression in applications such as drug repurposing. Networks are clear, understandable and flexible data structures on which associations can be implied using many statistical and computational approaches. The perception of interaction network is massively engaged in biology. In network models pairwise relations between various objects is exhibited. Schematically, in such networks, nodes are represented by entities (genes, proteins, complexes, metabolite, disease), while edges represent interactions or relationship between two nodes such the relationship between drugs and known gene targets and large number of diverse connections between two nodes can be displayed concurrently (Savva et al., 2019). Despite of the potency of such approaches has been verified for considerable times with drug-target interaction prediction, these methods are afflicted by the deficiency of current knowledge on molecular interactome, leading to noisy results. Network-based drug repositioning methods can be organized into categories based on their main source of biological data: 1) gene regulatory networks, 2) metabolic networks, and 3) drug interaction networks (Approaches et al., 2019). Moreover, a fourth category, integrated approaches, using multiple data sources simultaneously, can also be supplemented.

For example, a recent work proposed a untried bidirectional drug repositioning approach that comprised of Top-down and Bottom-up approaches and eventually provided information about significant repositioning drug candidates (Rakshit et al., 2015). This method takes into account tripartite indication-drug-target network (IDTN), also considering the topological significance (choosing most potent drugs based on seven topological parameters, such as degree, betweenness, centroid, closeness, eccentricity, radiality, and stress, which are basic network measures used to analyse a network) of drugs. A separate study proposed a different approach based on a two-pass random walk with restart on the drug-disease heterogeneous network, referred to as TP-NRWRH, to predict new indications for approved drugs (Liu et al., 2016). It was applied on three different types of networks, that is, integrated drug-drug similarity, disease-disease similarity, and drug-disease networks. This method was evaluated and in case study on the AD it showed that nine of top 10 predicted drugs have been approved or are investigational for neurodegenerative diseases.

#### 2.2.3 Genome-wide association studies-based methods

Another robust tool for drug repurposing is the utilization of genomics technologies. For the past few years, genome wide association studies (GWAS) has been another source of data which is being exploited for new information regarding the association of specific genomic variations known as single nucleotide polymorphisms (SNPs), with complex trait human diseases, such as AD, multiple sclerosis, etc. (Savva et al., 2019). GWAS can distinguish thousands of SNPs synchronously and these data are used by researchers to detect genes that are linked with a specific disease trait and to analyse how these variations affect responses to drugs. Furthermore, GWAS can be indicated to identify alternative indications for existing drugs rapidly and systematically (Hurle et al., 2013). However, objections such as inadequacy of data regarding whether an activator or inhibitor is needed to observe an effect, makes it burdensome to use GWAS information alone. While applying GWAS to initiate repurposing of drug candidates, the basic process is to analyse the catalogue of SNPs linked with the disease to determine a subgroup of genes that are speculated to be drug targets according to the drug ability of the gene’s product. Thereafter, process demands to select which of these gene products, if any, are targets for the drugs that are in the pharmaceutical channels at that instant. One such illustration detected by this approach is a clinical candidate Biib-033 (Biogen Idec, Cambridge, MA, USA), which is an antibody targeting the leucine-rich repeat and immunoglobulin domain-containing 1 (LINGO-1), which was developed for multiple sclerosis. Two GWAS studies detected LINGO-1 as a target for essential tremor, which is a neurological disorder, propounding that it could be repurposed for vital tremor ailments (Gudjonsdottir et al., 2009; Clark et al., 2010). .

## 3 Drug repurposing for neurodegenerative diseases

**FIGURE 2:**
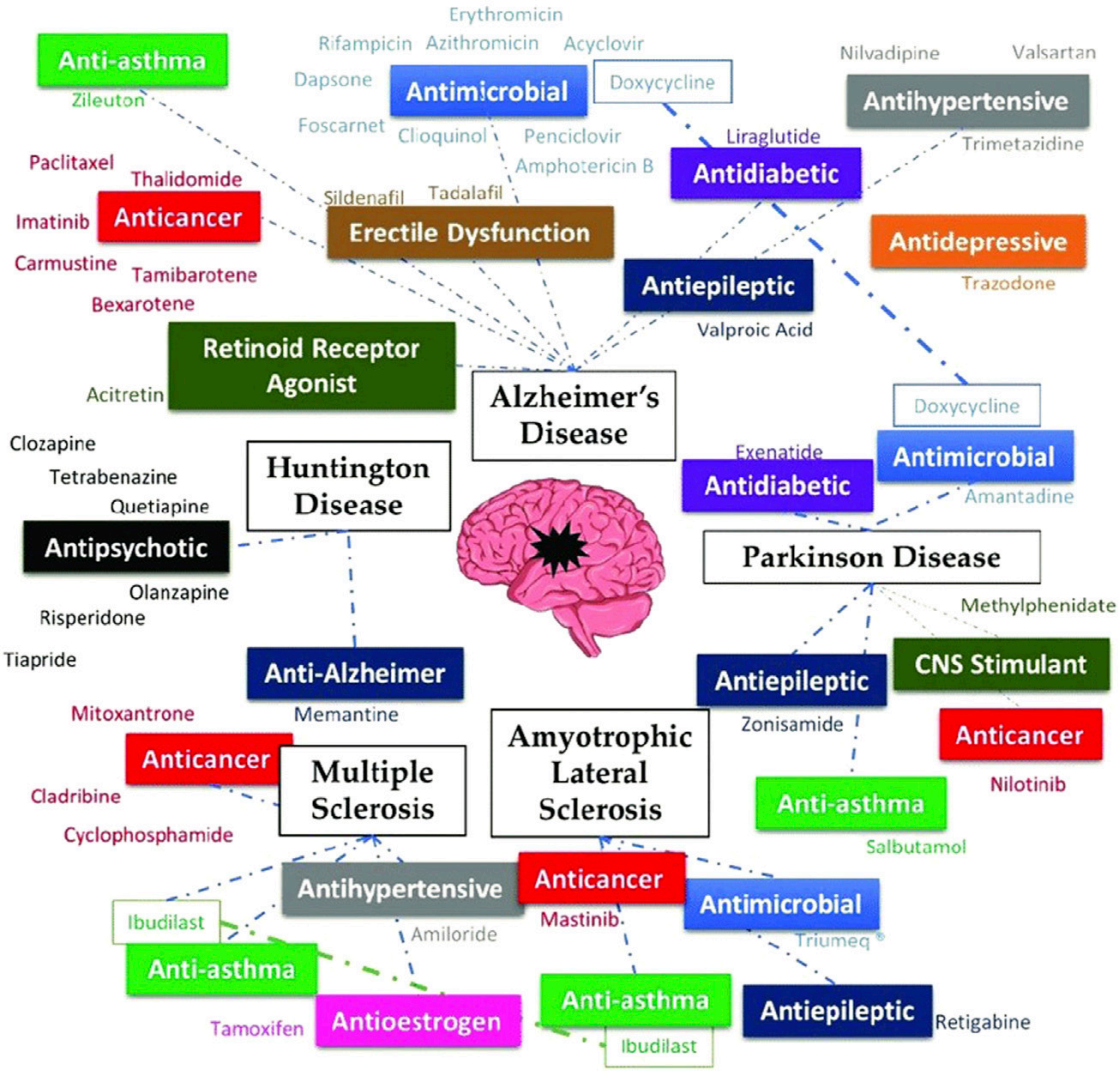
Summary of a few drugs repurposed for neurodegenerative diseases, adapted from (Durães et al., 2018) *via* CC by 4.0 license.

Diseases that affect the central as well as the peripheral nervous system, are known as neurodegenerative diseases (NDs). More than 600 distinct neuropathological illnesses exist, which include stroke, Parkinson’s disease, brain tumors, and epilepsy. Considering that the global population is growing, there are more NDs than ever before (Siuly and Zhang, 2016; Matilla-Dueñas et al., 2017; Kumar et al., 2021). In the next 20 years, neurodegenerative disorders that impact motor function will overtake cardiovascular disease as the second most common cause of mortality, according to the World Health Organization. No ND is currently curable due to its poorly understood molecular basis, and the medicines available merely treat the symptoms or slow the disease’s course (Onyango et al., 2021). Since the medicine’s pharmacokinetic and pharmacodynamic properties are already known, DR is the most beneficial new technique for the creation of an effective treatment for NDs. The promise of old medications for the most important NDs, like Amyotrophic lateral sclerosis, Huntington’s disease, Parkinson’s disease, Multiple sclerosis, and Alzheimer’s disease has been the subject of numerous studies (Durães et al., 2018). Figure 2 represents a summary of drugs repurposed for some neurodegenerative diseases.

### 3.1 Alzheimer’s disease

AD accounts for 80% of occurrences of dementia in senior persons. The gradual memory loss, the incapacity to learn, and the deterioration in behavior and function are its signs. Although the exact pathology of AD is unknown, it is thought to be related to the buildup of amyloid-β plaques in the brain, which eventually cause neuronal and synaptic degeneration (Scheltens et al., 2016). The majority of AD medications are used to address cognitive impairments or other symptoms, and they work best when started early (Appleby et al., 2013).

Commonly prescribed drugs for AD are cholinesterase inhibitors viz. Galantamine, Donepezil, Rivastigmine etc. Galantamine, an alkaloid found in *Galanthus* species, has been researched as a potential treatment for peripheral neuropathies and myopathies. It has the potential to block muscle acetylcholinesterase. Galantamine’s ability to improve nerve impulse transmission also makes it useful for reversing neuromuscular blockade during anesthesia. During the 1960s through the 1980s, the majority of galantamine use was confined to Italy, Bulgaria, Germany, and France under the brand name Nivalin®. Galantamine’s therapeutic properties for the treatment of AD were first investigated in the 1980s, and it was only in 2000 that it was included in the arsenal of drugs used to treat AD (Mucke, 2015). The production of misfolded proteins, oxidative stress, mitochondrial dysfunction, and impaired cell metabolism are only a few of the signaling pathways that may be involved in the pathogenesis of both cancer and neurodegeneration. The goal of the subsequent research was to see whether cancer medications may also be used to treat AD. Following these, investigations have been made to see if cancer medications can also be used to treat AD (Monacelli et al., 2017). Pathogens can enter the CNS in a variety of ways, depending on the organism, which may speed up the development of AD. The first is accomplished by a damaged BBB (Orgogozo et al., 2003). Some viruses, like the herpes virus, can go dormant after the original infection and then reawaken decades later in elderly people, causing delayed harmful complications (Nagarajan and Wilde, 2005). According to a 2020 study model, immunocompromised people who were exposed to *C. pneumoniae* through their noses developed Aβ plaque and NFTs in their olfactory cortex as well as in hippocampus (Sundar et al., 2020). Thus antimicrobials such as Rifampicin, Amphotericin B, acyclovir, penciclovir, foscarnet etc. (see Table 1) have also been researched to see whether they may be used to treat AD, specially its symptoms (Iqbal et al., 2020). Antidiabetics are also used to treat AD because type 2 diabetes has been established as a risk factor for the disease. According to studies, AD sufferers’ brains have become less sensitive to insulin signalling. Insulin therapy has been shown to improve memory and cognition while also protecting the brain from damage and controlling the levels of phosphorylated tau protein. Additionally, insulin can promote cell growth, repair, and activation of neural stem cells. As a result, substances that affect insulin release may potentially be beneficial for AD. Analogues of glucagon-like peptide 1, which increase insulin production, may also have an impact on a number of AD-related processes, including tau phosphorylation, amyloid-β reduction, and impaired neuronal function and cell death (Perry et al., 2003; Zhao et al., 2004). Some drugs repurposed for AD are listed in Table 1.

**TABLE 1:** List of repurposed drugs for AD.

Drug name	Earlier indication	Repurposed	References
Carmustine	It is a small, lipophilic, non-ionized nitrosourea molecule that can cross the blood-brain barrier and is employed as an alkylating agent in cases of brain cancer	Carmustine, at a non-toxic dose, demonstrated a significant reduction in amyloid-β development in cells overexpressing the precursor protein to the amyloid protein	Hayes et al. (2013)
Bexarotene	A retinoid X receptor antagonist is used to treat cutaneous T-cell lymphomas	In mice overexpressing familial AD mutations, it has been demonstrated to be effective at reversing neurodegeneration, enhancing cognition, and lowering amyloid-β levels	Tousi (2015)
Tamibarotene	It is an agonist of the retinoic acid receptor and is used to treat acute promyelocytic leukemia	It can influence a variety of pathways involved in the pathogenesis of AD, including those that control the release of pro-inflammatory chemokines and cytokines by brain cells, the behavior of animals with increased senescence, and cortical acetylcholine levels	Fukasawa et al. (2012)
Paclitaxel	It is an antimitotic drug authorized for the treatment of non-small cell lung cancer as well as ovarian and breast cancer	Although paclitaxel can be a substrate for P-gp and only penetrates a small portion of the central nervous system, it is particularly helpful in treating tauopathies because it reduces tau protein phosphorylation	Brunden et al. (2011)
Thalidomide	It prevents angiogenesis, endothelial cell growth, and blood-brain barrier disruption	Through the inhibition of tumor necrosis factor-α, it can minimize the death of hippocampus neurons	Ryu and McLarnon (2008)
Azithromycin, erythromycin	Macrolide antibiotics	They prevent the production of the amyloid precursor protein, which lowers the amyloid-β levels in the brain	Appleby et al. (2013)
Tetracyclines	Antibiotic (protein synthesis inhibitors)	It has been discovered that it encourages the destruction of fibrils and inhibits the synthesis of amyloid-β	Diomede et al. (2010)
Rifampicin	Use for *Mycobacterium* infections	It has shown results in the reduction of amyloid-β fibrils in a dose-dependent manner because of reduced production and enhanced elimination of amyloid-β	Tomiyama et al. (1996)
Acyclovir, penciclovir, foscarnet	antiviral drugs	In AD cell models, decreases phosphorylated tau protein and amyloid-β	Wozniak and Itzhaki (2010)
Amphotericin B	Antifungal drug	It has been demonstrated to slow down the production of amyloid-β (but posses toxicity)	Hartsel and Biochemistry (2003)
Clioquinol	Antifungal, Antiparasitic	In transgenic mice brains, it shows a reduction in the amyloid-β plaques	Grossi et al. (2009)
Valproic acid	Antiepileptic drug	Due to its ability to alleviate memory impairments and diminish the production of amyloid-β plaques in transgenic mice, it is recommended as a neuroprotective treatment for AD.	Smith et al. (2010)
Valsartan	Antihypertensive (angiotensin receptor blocker)	Chronic adverse stress, which can increase brain angiotensin II levels, is one of the main environmental factors of AD. Because it has been shown that angiotensin II increases are linked to amyloidogenesis, using angiotensin receptor blockers may be useful in delaying the loss of cognitive processing. Additionally, valsartan reduces inflammation, vasoconstriction, and mitochondrial dysfunction while encouraging acetylcholine release	Culman et al. (2002)
Trimetazidine	Anti-ischemic drug	It can penetrate the blood-brain barrier, lower free radical production, enhance axonal regeneration, and effectively myelinate both healthy and damaged axons	Hassanzadeh et al. (2015)
Liraglutide	Anti-diabetic drug	It demonstrated brain penetration and indicated physiological changes in the brain that improved learning and reduced the development of amyloid-β and inflammation in the brain	Mcclean et al. (2011)
Ghrelin	Peptide hormone (synthesized in the alimentary tract which controls appetite)	It has been shown that ghrelin, as well as its deacylated precursor, has neuroprotective effects by preventing programmed cell death and reducing the rise of interleukins induced by amyloid-β	Wagner et al. (2017)
Acitretin	Retinoid receptor activators	It reported an increase in antioxidant regulation and amyloid- β clearing enzymes	Tippmann et al. (2009)
Zileuton	Antiasthma drug	Zileuton, which inhibits 5-lipoxygenase, is thought to offer therapeutic benefits for AD. This is due to the finding that 5-lipoxygenase is more prevalent in AD, creating it an exciting target within this context. Research using zileuton in mice revealed a decrease in amyloid-β accumulation	Di Meco et al. (2014)
Sildenafil/tadalafil	Erectile dysfunction drugs (inhibitors of phosphodiesterase-5) Phosphodiesterase-5 regulates cGMP, which in turn regulates memory problems caused on by amyloid-β	In aged mouse models, sildenafil was effective in reducing amyloid-β and suppressing neuroinflammation. Furthermore, Tadalafil showed neuroprotection and an increase of cognition	García-Barroso et al. (2013), Zhang et al. (2013)
Trazodone	Antidepressant	Trazodone has demonstrated potential in suppressing signaling *via* the PERK/eIF2α-P branch of the unfolded protein response, which is overactivated in AD patients and harms regulating translation s in cells	Halliday et al. (2017)

### 3.2 Parkinson’s disease

PD is a multifactorial neurological condition that impairs a patient’s ability to move. Dopamine neurons in the putamen and caudate areas of the brain are the main targets of Parkinson’s disease. Due to mitochondrial DNA deletion, elevated ROS and RNS generation decreased antioxidant function, and dopamine inhibition, the activities of mitochondria are reduced in the substantia nigra of parkinsonian brains (Ryan et al., 2015; Reeve et al., 2018). As dopamine is oxidized by both Monoamine oxidase (MAO) A and B, the level of dopamine drops in PD (Alexander, 2004). Primary tremor, akinesia, rigidity, bradykinesia, lack of postural instability, and secondary motor symptoms including the freezing of gait, micrographia, and speech issues are the hallmarks of Parkinson’s disease (PD). In PD, non-motor symptoms include sensory impairment, autonomic dysregulation, neurobehavioral abnormalities, and sleep problems are also possible. Parkinson’s disease is treated with levodopa, carbidopa, amantadine, rotigotine, dopamine agonists, Catechol-O-methyltransferase (COMT) inhibitors, anticholinergics Selegiline, rasagiline, safinamide, etc (Gupta and Shukla, 2021). The most recent therapy options for PD include newer dopaminergic medications, immunotherapies, drug repurposing, medications that target non-dopaminergic neurotransmitters, regenerative treatments, and deep brain stimulation. Many medications are currently undergoing clinical trials. Several medications, including the following, are being repurposed for PD: The antibiotic doxycycline, which has been investigated for its anti-PD effects after being once identified as a possible anti-AD therapeutic approach (Dominguez-Meijide et al., 2021). Differences in doxycycline concentration can distinguish between an antibacterial and an anti-inflammatory effect. Smaller concentrations than the ones used to treat microorganisms with antibiotics do not influence bacterial susceptibility, according to studies, but they do exhibit anti-inflammatory activity, which is connected to their neuroprotective effects. Doxycycline’s antioxidant properties and its capacity to transform early species of α-synuclein oligomers (a presynaptic neuronal protein connected to PD genetically and neuropathologically) into non-toxic and non-seeding species are two additional ways that aid neuroprotection (Dominguez-Meijide et al., 2021). Only oligomeric species of α-synuclein have been discovered to bind to doxycycline, however, the physiological monomeric forms of α-synuclein are still present. Table 2 represents repurposed drug for PD. The anti-PD activity of antiasthma medications, specifically β2-adrenoreceptor agonists, has been researched. Recent research has connected the β2-adrenoreceptor to the control of the SNCA-synuclein gene. More particular, stimulation of the β2-adrenoreceptor was demonstrated to exhibit neuroprotection. Three anti-asthmatic drugs were investigated, and salbutamol, the one with the highest blood-brain barrier permeability, demonstrated the greatest promise. The conducted analysis revealed that all three medications were capable of lowering the abundance of SNCA-mRNA and α-synuclein (Mittal et al., 2017).

**TABLE 2:** List of repurposed drugs for PD.

Drug name	Earlier indication	Repurposed	References
Amantadine	Anti influenza	As a mild glutamate receptor antagonist, it is used to treat Parkinson’s disease (PD), boosting dopamine and preventing its reuptake	Lee and Kim, (2016)
Nilotinib	Tyrosine kinase Abl inhibitors, used to treat chronic myeloid leukaemia	It was found that α-synuclein build-up and increased α-synuclein expression are both signs of Abl activation in neurodegeneration. Nilotinib accelerates α-synuclein breakdown by preventing Abl phosphorylation	Pagan et al. (2016)
Zonisamide	Antiepileptic drug	Increased dosages revealed a reduction in intracellular dopamine. Both motor and non-motor symptoms have responded well to this medication, but its exact mode of action is yet unknown	Fox et al. (2018)
Methylphenidate	Central nervous system stimulant used to treat attention-deficit hyperactivity disorder	This medication has been found in numerous studies to be beneficial in lowering PD-related gait problems and non-motor symptoms	Devos et al. (2013)
Exenatide	Glucagon-like peptide-1 (used for type 2 diabetes)	It has proven to be capable of neuroprotection and beneficial neuroplastic change, which can stop or reduce the progression of the disease. It can cross the blood-brain barrier and offers neuroprotection by turning on GLP-1 receptors	Jankovic, (2017)

### 3.3 Huntington’s disease

HD is characterized by dementia, behavioral and mental abnormalities, and involuntary choreatic movements (McColgan and Tabrizi, 2018). The multifunctional protein huntingtin (HTT) develops a mutant form as a result of a genetic mutation, which causes toxicity and causes neuronal death and malfunction. When a mutation in the HTT gene’s exon 1 on chromosome 4p16.3 results in CAG (C-cytosine, A-adenine, and G-guanine) trinucleotide DNA segment extension, repetition, and multiplicity, HD develops. In a gene, the CAG segment is typically repeated between 10 and 35 times. However, due to mutations, more than 36 CAG repeats are produced, which results in the genesis of HD (Tabrizi et al., 2020). The slow degeneration of neurons in the basal ganglia, particularly the caudate nucleus and putamen to the cerebral cortex, signals the beginning of HD (Kshirsagar et al., 2021). The symptoms of HD begin to appear in adults, and they worsen with time until they eventually result in death within years. The sole alternative is to control the symptoms since there is no known cure for this illness s (Roos, 2010).

Tetrabenazine was initially created as a result of research into the design of straightforward drugs with reserpine-like antipsychotic action. It functions as both a mild blocker of the D2 dopamine postsynaptic neurons and a highly selective, reversible inhibitor of monoamine absorption by presynaptic neurons. Research on this substance as an antipsychotic was conflicting, thus this medication was repurposed for conditions like HD that are characterized by abnormal, involuntary hyperkinetic movements. Tetrabenazine has never been shown to elicit signs of dyskinesia, making it a safer drug to use in HD than dopamine receptor blockers (Paleacu, 2007). For the treatment of HD, several medications with dopamine antagonistic action have been investigated. This is the situation with the antipsychotic drug tiapride, a D2 receptor antagonist. Selegiline, however, is a popular option for the treatment of Huntington’s chorea in Europe (Roos et al., 1982). A neuroleptic medication called clozapine is used to treat schizophrenia. With little antagonistic activity toward the D2 dopaminergic receptors, it exhibits a high affinity for the D1 and D4 dopamine receptors. Although clinical trials had mixed outcomes, it was recommended as a good symptomatic medication for chorea due to its low prevalence of extrapyramidal side effects (Bonuccelli et al., 1994). Another antipsychotic medicine, olanzapine, is frequently recommended to treat HD’s behavioral and motor symptoms. While antagonizing dopamine D2 receptors, this medication has a high affinity for serotonin receptors. It can be advised when irritation, sleep issues, weight loss, and chorea are present because it is safe and well tolerated (Paleacu et al., 2002). As a D2 receptor antagonist and serotonin agonist, the antipsychotic risperidone, which is used to treat schizophrenia and bipolar disorder, can also be used to treat HD chorea. It demonstrated positive results in stabilizing mental symptoms and motor deterioration (Duff et al., 2008). Quetiapine, an atypical antipsychotic, has a strong affinity for dopamine and serotonin receptors. Even though there haven't been many instances of quetiapine being used to treat HD symptoms, those have emphasized the drug’s value in treating chorea, particularly when it's coupled with psychiatric symptoms (Alpay and Koroshetz, 2006). An adamantane derivative called memantine is used to treat AD. It is an inhibitor of N-methyl-D-aspartate (NMDA) that is non-competitive. A large influx of calcium enters the cell as a result of excessive NMDA receptor stimulation, which ultimately results in cell death. Memantine can therefore stop this calcium influx in neuronal cells and stop the death of brain cells. When memantine’s effectiveness in treating HD was investigated, it was shown that it could lessen the susceptibility of neurons to glutamate-mediated excitotoxicity (Beister et al., 2004). Table 3 represents list of repurposed drugs for HD.

**TABLE 3:** List of repurposed drugs for HD.

Drug name	Earlier indication	Repurposed	References
Clozapine	Neuroleptic drug	Although clinical trials had mixed outcomes, it was recommended as a good symptomatic medication for chorea due to its low prevalence of extrapyramidal side effects	Bonuccelli et al. (1994)
Tetrabenazine	Intended to have antipsychotic effects but produced conflicting success	Repurposed to treat HD symptoms, it functions as a mild blocker of D2 dopamine postsynaptic neurons and a high-affinity, reversible inhibitor of monoamine uptake by presynaptic neurons	Paleacu (2007)
Olanzapine	Antipsychotic drug	It is routinely prescribed for the treatment of HD’s motor and behavioural symptoms. Although this medication has a strong affinity for serotonin receptors, it is antagonistic to dopamine D2 receptors	Paleacu et al. (2002)
Risperidone	Antipsychotic drug	It is used to treat schizophrenia and bipolar disorder as a D2 receptor antagonist and serotonin agonist, and it can also be used to treat HD chorea	Duff et al. (2008)
Memantine	Used to treat AD.	Investigation into memantine’s efficacy for treating HD revealed that it could lower neurons’ sensitivity to glutamate-mediated excitotoxicity	Beister et al. (2004)

**TABLE 4:** Some repurposed drugs for ALS and MS.

Drug name	Earlier indication	Repurposed	References
Masitinib	Tyrosine kinase inhibitor (used to treat canine cancer)	Tyrosine kinase inhibitors may be effective against the aberrant glial cells that grow in ALS, explaining their usage in the disease	Trias et al. (2016)
Triumeq® (dolutegravir + abacavir + lamivudine)	An antiretroviral Drug used in anti-HIV therapy	Based on the fact that ALS patients had reverse transcriptase blood concentrations comparable to HIV-infected patients and that a human endogenous retrovirus was found to be expressed in the brains of ALS victims, this medicine was investigated for the treatment of the disease	Clinicaltrials (2022)
Retigabine	Anti-epileptic drug (causes membrane hyperpolarization by attaching to voltage-gated potassium channels, which increases the M-current.)	Because it is believed that neurons in this condition are hyper-excited and fire more frequently than usual, ultimately leading to cell death, it can promote motor neuron survival and lower excitability, which is beneficial in the treatment of ALS.	Wainger et al. (2021)
Tamoxifen	An antioestrogen drug (authorized for use in breast cancer chemotherapy and chemoprevention)	The discovery of neurological improvements in patients and disease stability in ALS patients who had breast cancer treated with tamoxifen led to the drug’s accidental repurposing for the treatment of ALS.	Chen et al. (2020)
Mitoxantrone	An anthracenedione that has been proven effective in the treatment of breast and prostate cancer, acute leukaemia, and lymphoma	Mitoxantrone has also been licensed for the treatment of MS due to its immunosuppressive properties, which are connected to variable responses of the T- and B-cells in the central nervous system to antigens, myelin degradation brought on by macrophages, and axonal lesions	Fox (2004)
Cyclophosphamide	An alkylating agent treatment of leukaemia, lymphomas, and breast carcinoma	Cyclophosphamide is used in MS because it can have an immunosuppressive and immunomodulatory effect. Additionally, cyclophosphamide has good absorption in the central nervous system and can cross the blood-brain barrier	Awad and Stue (2009)
Amiloride	A diuretic medication	Amiloride can prevent the neuronal proton-gated acid-sensing ion channel 1 (ASIC1), which is overexpressed in axons and oligodendrocytes in MS lesions, from having its neuroprotective and myeloprotective effects. A further benefit of amiloride’s preventive action occurring later in the course of inflammation is that it makes it active even before inflammation begins	Arun et al. (2013)
Ibudilast	Phosphodiesterases inhibitor used for bronchial asthma and cerebrovascular disorders	Ibudilast can prevent the brain’s microglia and astrocytes from releasing tumor necrosis factor, which reduces neuronal degeneration. It is also helpful in MS because it can prevent oligodendrocyte apoptosis, suppress astrocyte apoptosis, and prevent demyelination	Barkhof et al. (2010)

### 3.4 Other neurodegenerative diseases

Upper and lower motor neurons, which regulate the voluntary muscles, die as a result of the condition known as ALS. Muscles eventually weaken and shrink as a result, which causes muscular atrophy. Other signs include difficulty breathing, swallowing, speaking, and twitching or rigid muscles. Most ALS causes are aetiologically unknown, with genetic inheritance accounting for roughly 10% of cases (Kiernan et al., 2011). Only two medications, edaravone, and riluzole, are presently accessible to postpone the development of the illness, albeit they cannot reverse the symptoms once they have appeared (Zoccolella et al., 2007; Sawada, 2017). Another autoimmune condition affecting the central nervous system is MS. It is a protracted, inflammatory disorder in which the myelin and axons are partially or completely damaged. Its progression is uncertain, and its early symptoms include temporary neurological impairments that eventually turn severe. There is currently no approved treatment for MS, however, there are medications that can slow the disease’s progression and symptoms (Trapp and Nave, 2008). Several drugs are currently being repurposed for the treatment of ALS as well as MS. Table 4 represents some drugs that are under clinical trial for ALS or MS.

### 3.5 Unsuccessful repurposed drugs for neurodegenerative diseases

Even though there have been numerous instances of pharmacological repurposing, numerous attempts at repositioning have also been unsuccessful. A drug may look promising in computational analyses or *in vitro* assays but not *in vivo*, requiring the investigation of the medicine to be stopped in favor of other activities. This was the situation with latrepirdine, an antihistamine that was repurposed for AD and HD after being licensed in Russia for the treatment of rhinitis brought on by allergies. Despite the lack of a characterized mechanism of action, it had been suggested that it might alter the activity of channels and neurotransmitters, avoiding amyloid toxicity among other things (Bezprozvanny, 2010). In actuality, phase III studies unsuccessful to find any appreciable variations in the course of the disease, despite phase II research showing improvement in AD patients related to placebo (Doody et al., 2008). There have also been attempts to employ the anti-hypercholesterolemic medications simvastatin and atorvastatin for AD. This notion was developed in response to the important finding that cardiovascular illness and AD frequently co-occur. Studies had demonstrated that statins could raise neuroprotection and reduce amyloid-β levels, among other positive benefits. However, none of them were effective in the management of AD (DL et al., 2005; Sano et al., 2011). Studies evaluating the use of selective serotonin reuptake inhibitors, commonly used as antidepressants, in the treatment of AD have also been conducted. Although nortriptyline and paroxetine originally showed an improvement in cognitive abilities, subsequent analyses revealed that there was no improvement in cognitive behavior even after these medications had addressed mood disorders (Nebes et al., 2003).

In phase II investigations for the treatment of ALS, the antibiotic ceftriaxone seemed promising, but it also failed to demonstrate clinical efficacy in phase III tests (Cudkowicz et al., 2014). Even cladribine was initially rejected as an MS treatment before it was approved (Leist and Weissert, 2011). Even though DR is encouraging in the creation of new treatments for ND, the approval procedure can be challenging and frequently leads to the failure of repurposing initiatives.

## 5 Opportunities and challenges of drug repurposing

Owing to its proficiency in sparing time and cost, drug repositioning has become a crucial method for exploiting new therapeutic implications of current drugs or drug candidates. Such an ingenious type of approach will undeniably accelerate the drug development process. Concurrently, in the case of neurological diseases, some restraints need to be considered during the process of drug repositioning. Repurposing drugs experiences humongous challenges due to which there are limitations in the market for repurposed drugs. A single phase III clinical trial of a repurposed drug for AD can cost up to 300–400 million dollars (Shineman et al., 2014). This demonstrates even though repurposed drugs can deviate from the initial development stage and safety testing, they demand profound high-risk extravagant clinical trials to establish efficacy. As a result of the sluggish progression of neurodegenerative diseases, clinical trials might take a long duration. Further, apart from proving the drug penetration into the brain, many times drugs must be tested for safety issues in geriatric populations who periodically have comorbidities and undergo treatment that may interreact with the repurposed drug. DR may be difficult in neuropathological states considering its complex molecular and cellular signaling mechanisms. The reason that drugs respond to multiple targets despite affecting a single target might accelerate the risk of a range of adverse reactions (Vogt and Mestres, 2010). An all-inclusive evaluation of the assets as well as lacking these adverse effects can assist us to figure out drug repositioning from a more multifaceted perspective (Reddy and Zhang, 2013). Other challenges in repurposing drugs include limited or no patent protection or patent life, commercialization, and reimbursement challenges.

In pursuance of overcoming obstructions encountered in the course of drug repurposing, we can consider several proposals. In the first place, it is inspirited to furnish more financial support in conjunction with technical assistance for clinical trials of drugs to be repurposed. Pharmaceutical companies are exceedingly doubtful to finance human trials of approved drugs to be repositioned unless there is a viable commercial strategy. This generates a favorable circumstance where government and foundations can take the eagerness to do something. Currently, several groups are taking a large interest in funding pilot trials of repurposed drug candidates with the hopes of paying more impetus to drug repurposing. Foundations such as ‘Cures Within Reach’ are entirely centralized on aiding repurposing studies. The MJFF, ADDF, Cure Parkinson’s Trust, Alzheimer’s Society (United Kingdom), the Multiple Myeloma Research Foundation, and others have financed repurposing trials. In association with government initiatives, various academic centers are also heading the repurposing attempts. Secondly, to augment data sharing it is crucial to constitute an exhaustive data analysis platform. The enormous volume of data piled up by approved drugs or drug candidates for clinical trials can be stored in an assorted manner and can be unlocked and reanalyzed adopting Information science services and artificial intelligence. The bottleneck in the research process is that data derived from biological databases and human trials are massive and perplexed and the conventional data processing methods cannot work out with it. We can unquestionably improve our understanding of the disease from this big data and make more accurate disease-related strategies. Nevertheless, there is a considerable breach between producing biomedical data and data analysis. Expertise needs to find technical clarifications to ensure the efficiency of research with less energy and time. Finally, it is fundamental to resolve patent restrictions and take judicious surveillance in pursuance of facilitating the DR process. The utilization of drug reprofiling should be backed by a risk handling strategy and the drug’s safety assurance can be established by clinical trial information or data from post-marketing surveillance.

## 6 Conclusion

In recent years, many repurposed drugs have found their place as potential agents to treat various neurodegenerative diseases. As already discussed many companies are developing and elaborating the strategic advantages of using Artificial intelligence (AI) and machine learning (ML) based frameworks for drug discovery in this segment. Despite these advancements, there are threats to the precise analysis of existing pre-clinical and clinical evidence concerning particularly from regulatory and scientific perspectives. Apart from focussing on the efficacy of the newly repurposed drugs, robust post-authorization studies are equally important.

## Abbreviations

AD, Alzheimer’s disease; AI, artificial intelligence; ALS, amyotrophic lateral sclerosis; CMap, connectivity map; COMT, catechol-o-methyltransferase; DR, drug repurposing; EMR, electronic medical record; FDA, U.S. food and drug administration; FTD, frontotemporal dementia; HD, huntington’s disease; MAO, monoamine oxidase; ML, machine learning; MS, multiple sclerosis,; NDs, neurodegenerative diseases; OMOP, observational medical outcomes partnership; PD, Parkinson’s disease
